# Supplementary material for: Microstructure and Morphology Control of Potassium Magnesium Titanates and Sodium Iron Titanates by Molten Salt Synthesis
Source: Materials (Basel). 2019 May 14;12(10):1577. doi: 10.3390/ma12101577 (PMC6566462; doi:10.3390/ma12101577)
Supplement: Supplementary file 1 [file materials-12-01577-s001.pdf]

materials

Microstructure and Morphology Control of Potassium Magnesium Titanates and Sodium Iron Titanates by Molten Salt Synthesis

Haoran Zhang †, Mengshuo Li †, Ze Zhou, Liming Shen * and Ningzhong Bao *

Supplementary materials:

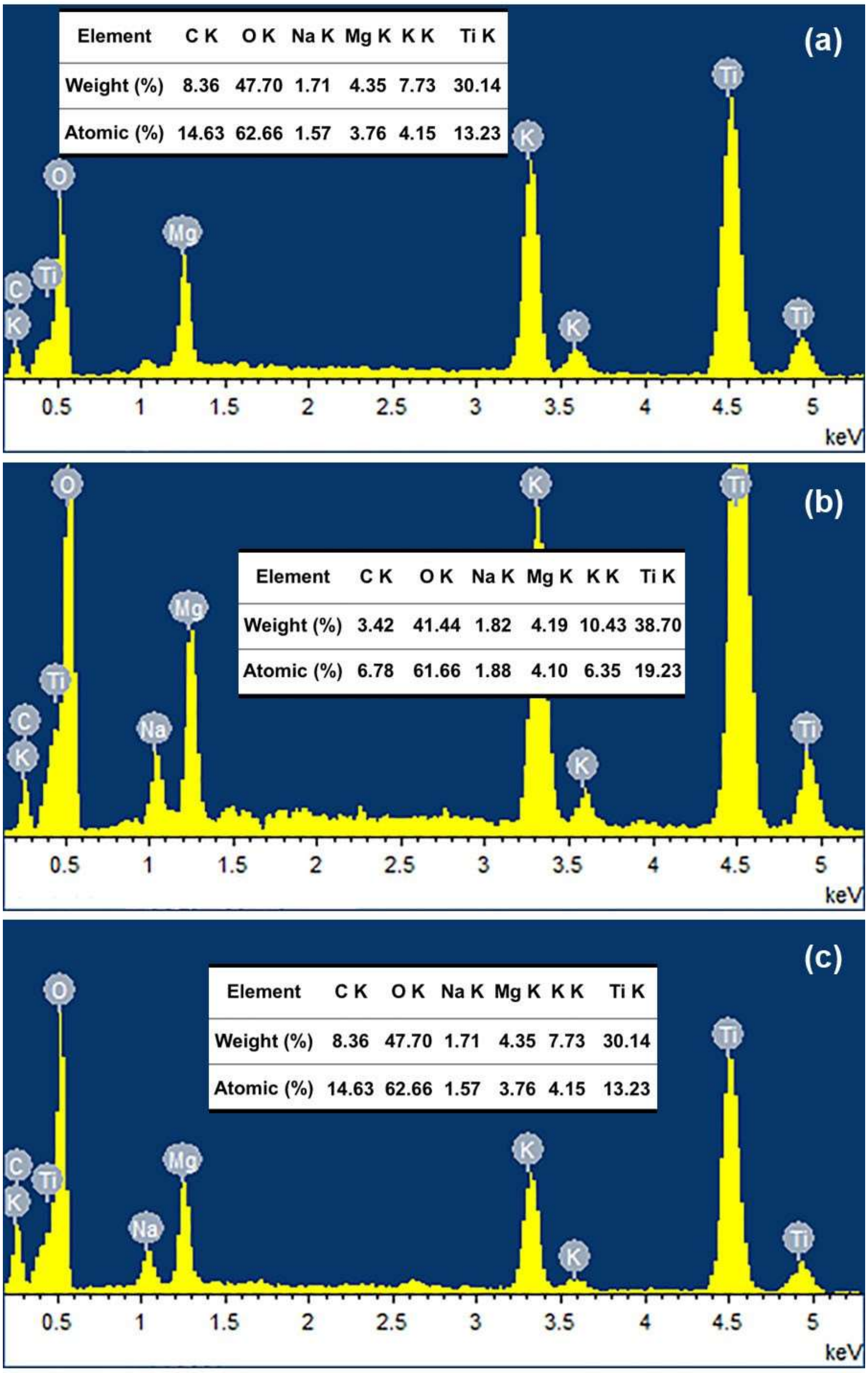

Figure S1. EDS analyses of (**a**) $K_{0.8}Mg_{0.4}Ti_{1.6}O_4$ platelets, (**b**) $K_{0.8}Mg_{0.4}Ti_{1.6}O_4$ boards, and (**c**) $K_{0.8}Mg_{0.4}Ti_{1.6}O_4$ bars.

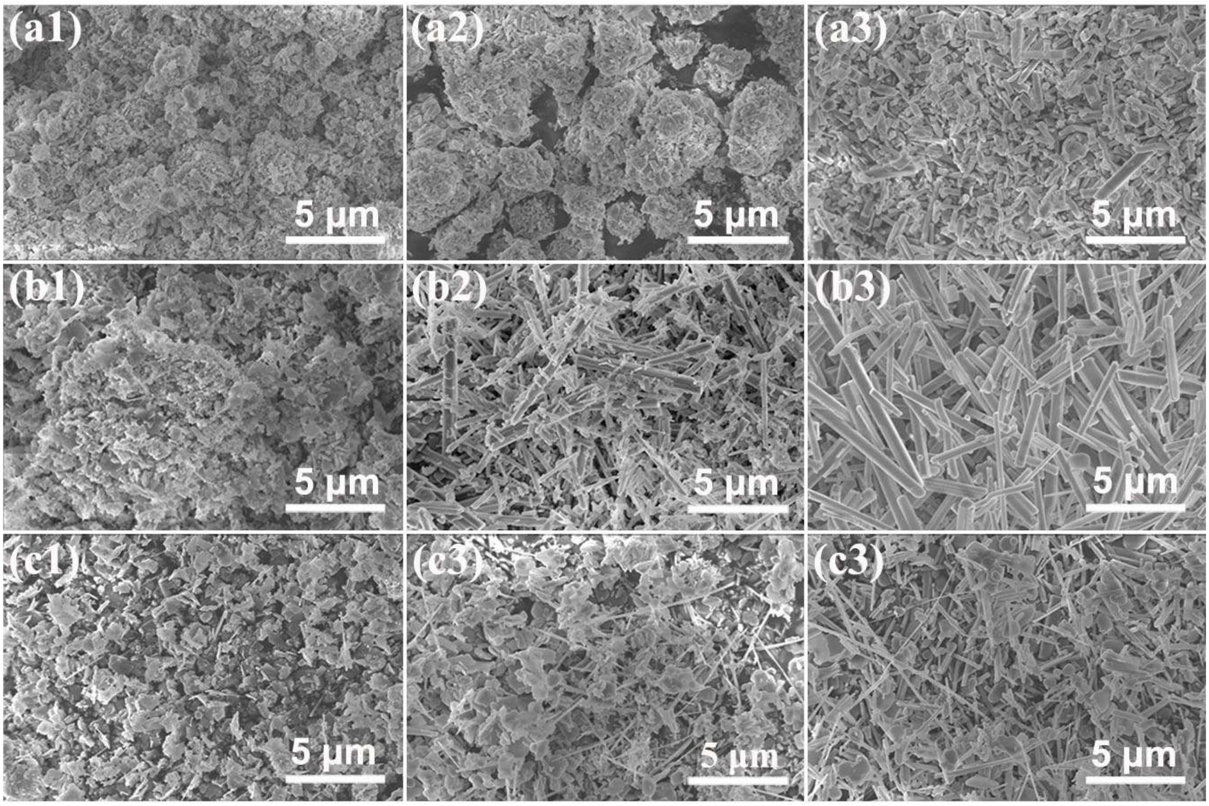

Figure S2. SEM images of the products obtained after annealing the starting materials for (a1–a3) $K_{0.8}Mg_{0.4}Ti_{1.6}O_4$ platelets, (b1–b3) $K_{0.8}Mg_{0.4}Ti_{1.6}O_4$ boards, and (c1–c3) $K_{0.8}Mg_{0.4}Ti_{1.6}O_4$ bars at different temperatures. (**a1, b1, c1**) 750 °C, (**a2, b2, c2**) 850 °C, and (**a3, b3, c3**) 950 °C.

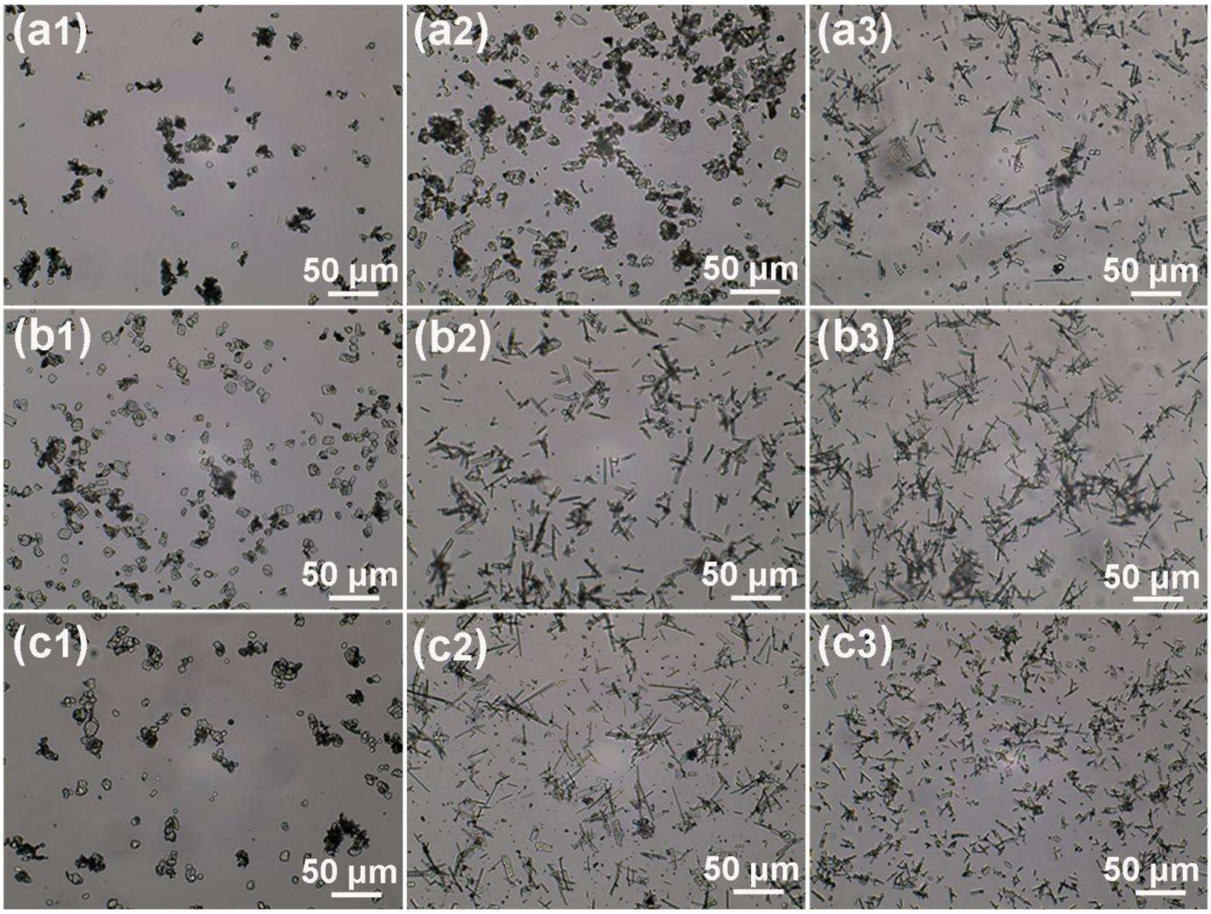

Figure S3. SEM images of the products obtained after annealing the starting materials for (a1–a3) $K_{0.8}Mg_{0.4}Ti_{1.6}O_4$ platelets, (b1–b3) $K_{0.8}Mg_{0.4}Ti_{1.6}O_4$ boards, and (c1–c3) $K_{0.8}Mg_{0.4}Ti_{1.6}O_4$ bars at different times. (**a1, b1, c1**) 2 h, (**a2, b2, c2**) 4 h, and (**a3, b3, c3**) 6 h.

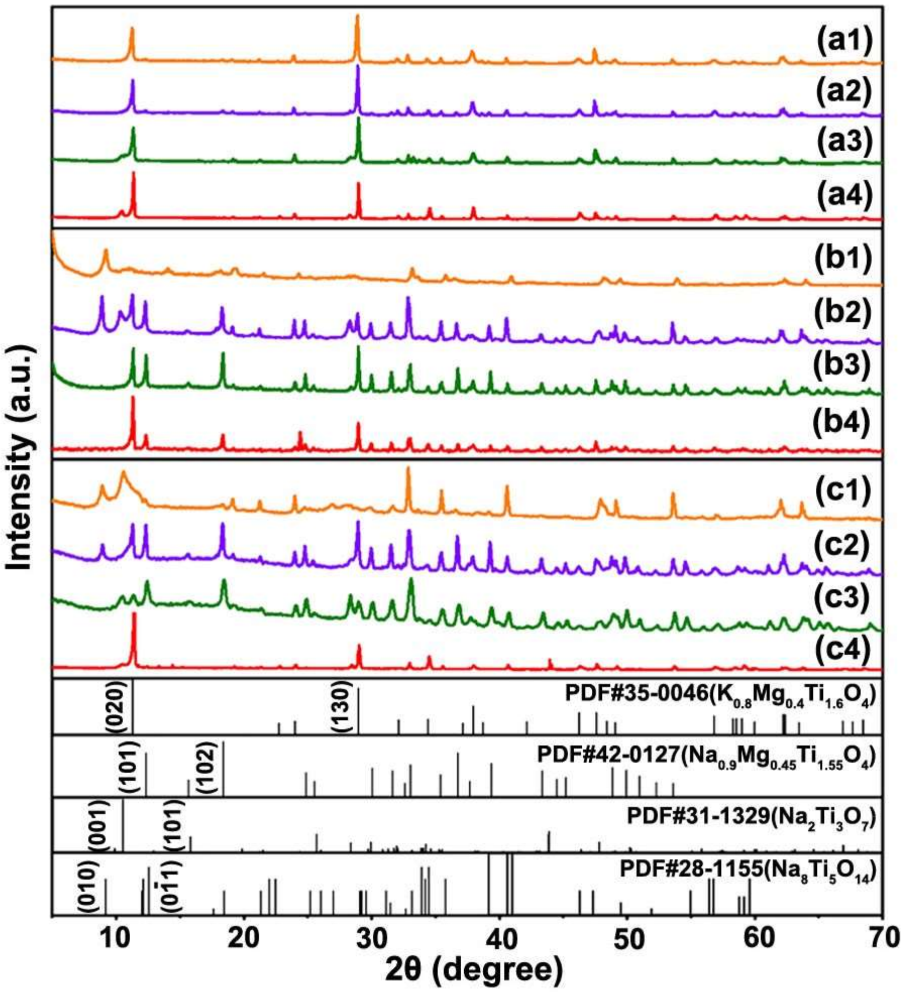

Figure S4. XRD patterns of the products obtained after annealing the starting materials for (a1–a4) $K_{0.8}Mg_{0.4}Ti_{1.6}O_4$ platelets, (b1–b4) $K_{0.8}Mg_{0.4}Ti_{1.6}O_4$ boards, and (c1–c4) $K_{0.8}Mg_{0.4}Ti_{1.6}O_4$ bars at different temperatures. (**a1, b1, c1**) 750 °C, (**a2, b2, c2**) 850 °C, (**a3, b3, c3**) 950 °C, and (**a4, b4, c4**) 1050 °C.

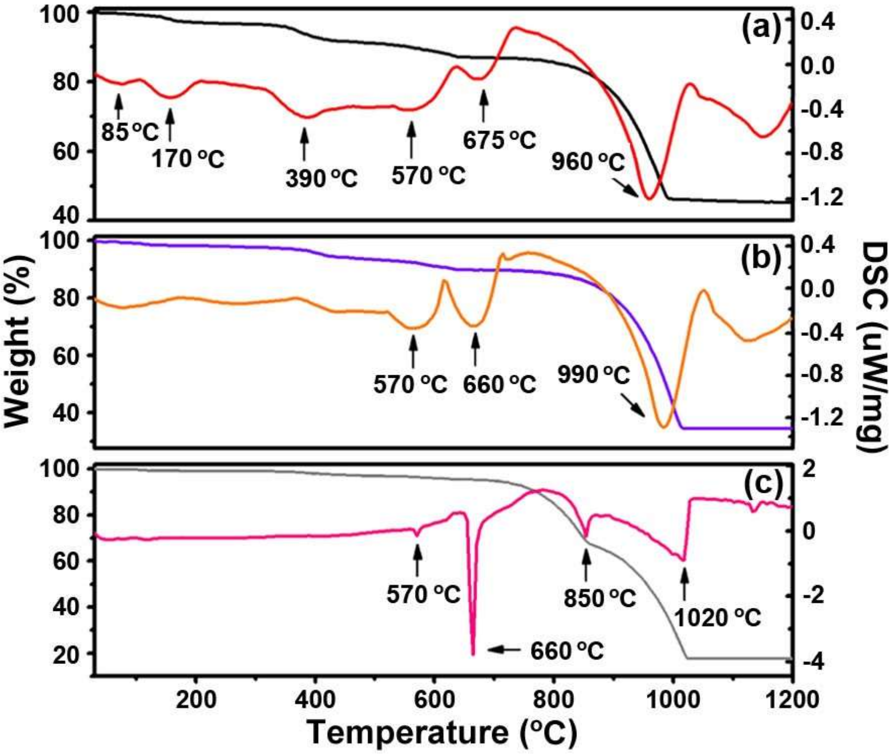

Figure S5. TG-DSC plots of (**a**) $K_{0.8}Mg_{0.4}Ti_{1.6}O_{4}$ platelets, (**b**) $K_{0.8}Mg_{0.4}Ti_{1.6}O_{4}$ boards, and (**c**) $K_{0.8}Mg_{0.4}Ti_{1.6}O_{4}$ bars.

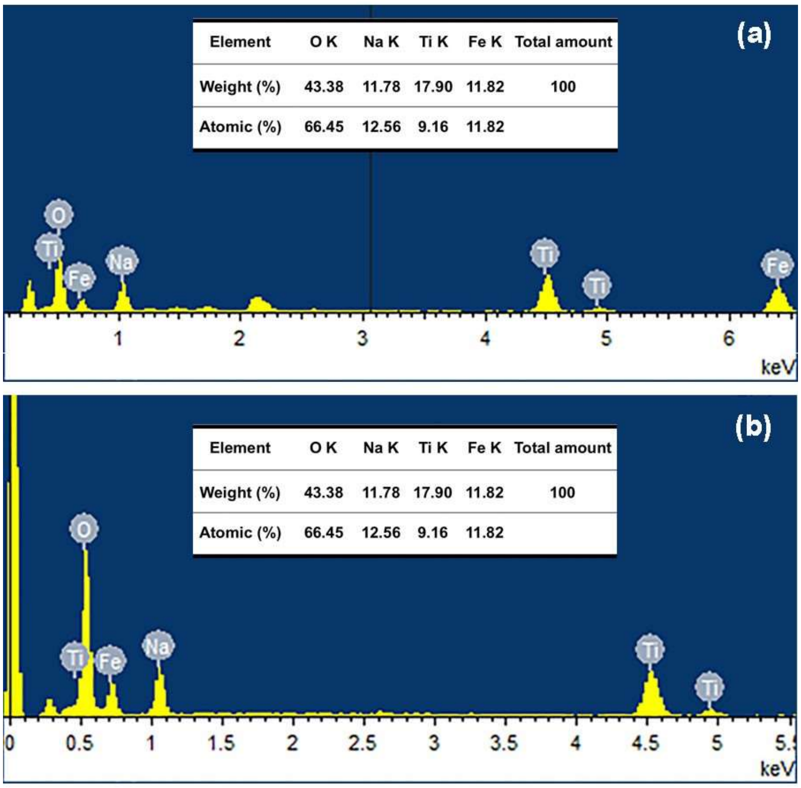

Figure S6. EDS analyses of (**a**) $K_{0.8}Mg_{0.4}Ti_{1.6}O_{4}$ platelets, (**b**) $K_{0.8}Mg_{0.4}Ti_{1.6}O_{4}$ boards, and (**c**) $K_{0.8}Mg_{0.4}Ti_{1.6}O_{4}$ bars.

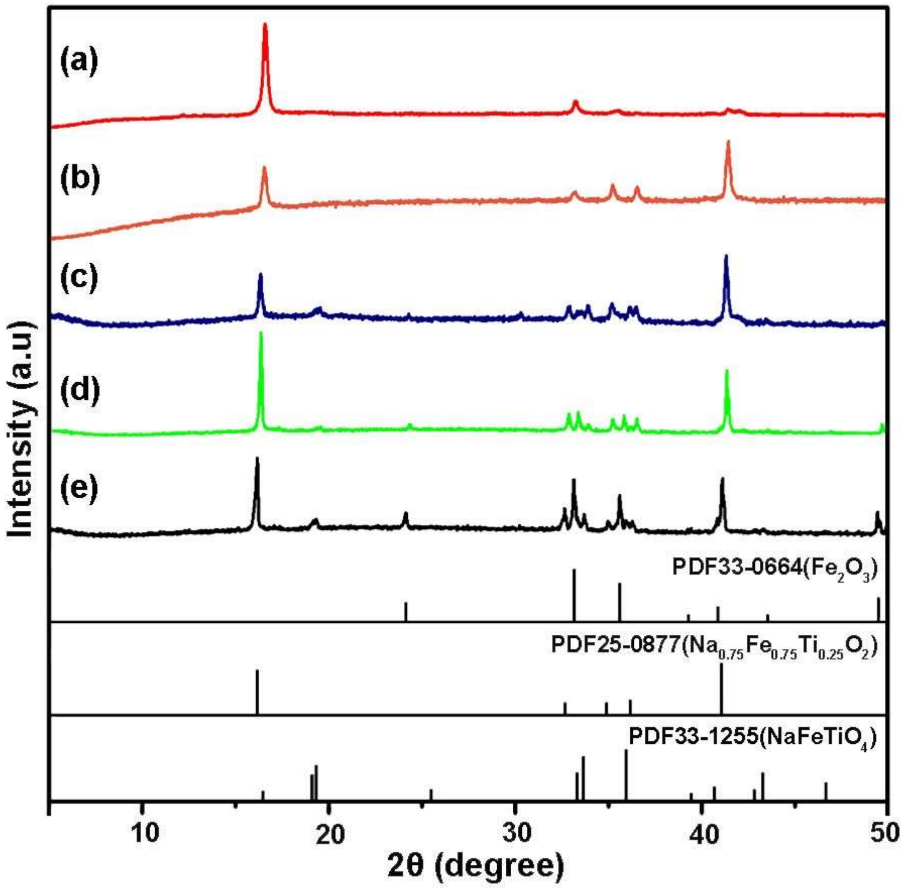

Figure S7. XRD patterns of the products prepared with different ratio of reactants for $Na_{0.75}Fe_{0.75}Ti_{0.25}O_2$. Na:Fe:Ti = (**a**) 3.3:1.5:1, (**b**) 3.3:2.0:1, (**c**) 3.3:2.5:1, (**d**) 3.3:3.0:1, and (**e**) 3.3: 3.5:1.

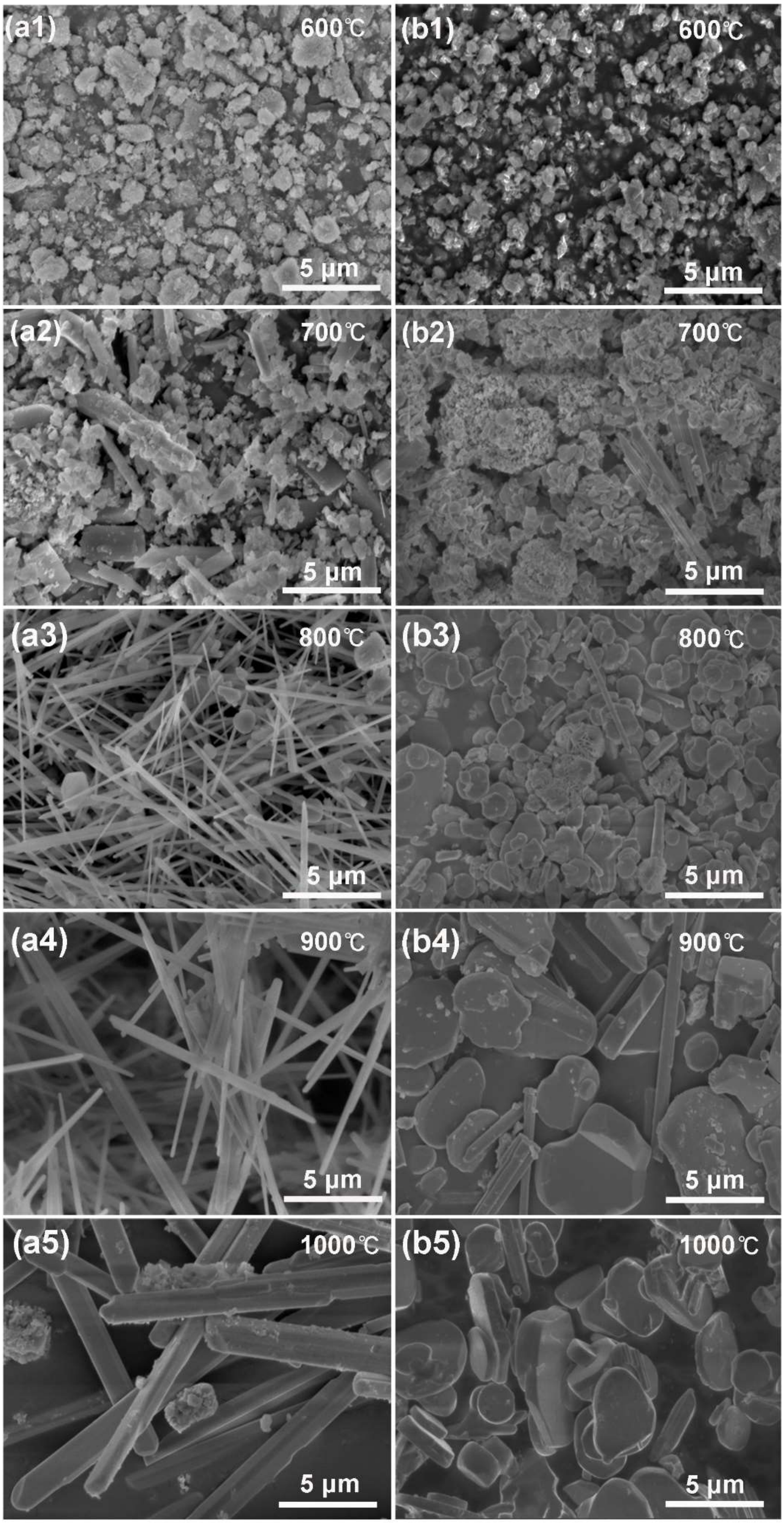

Figure S8. SEM images of the products obtained after annealing the starting materials for (a1–a5) $NaFeTiO_4$ and

(b1–b5) $Na_{0.75}Fe_{0.75}Ti_{0.25}O_2$ at different temperatures. (**a1, b1**) 600 °C, (**a2, b2**) 700 °C, (**a3, b3**) 800 °C, (**a4, b4**) 900 °C, and (**a5, b5**) 1000 °C.

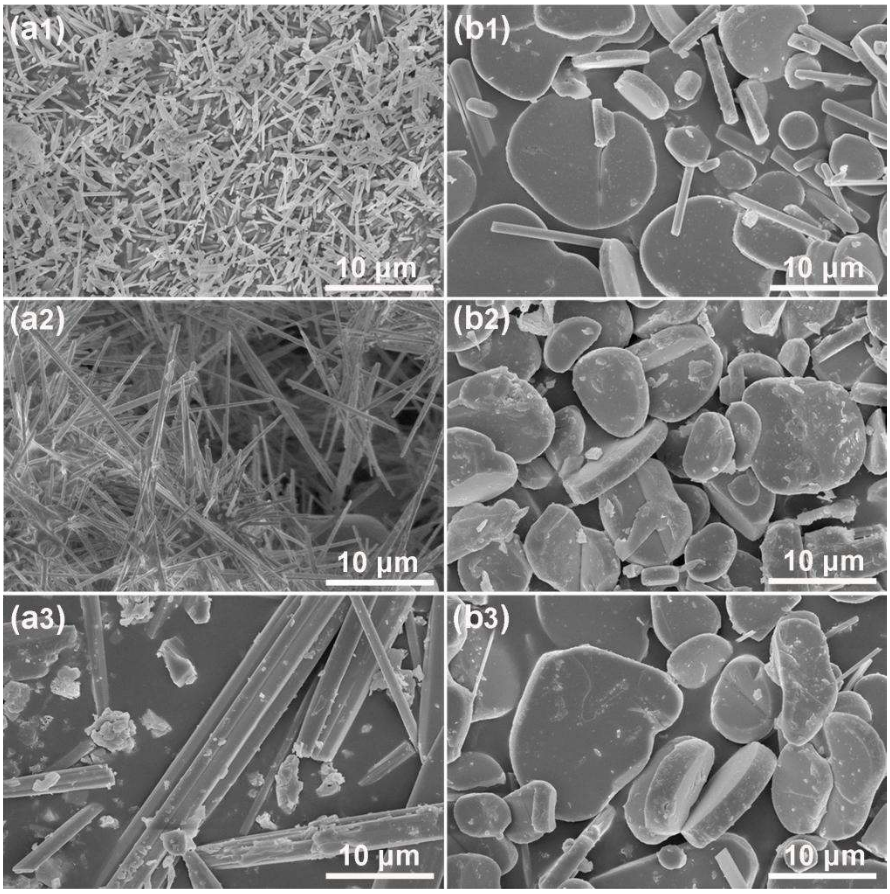

Figure S9. SEM images of the products obtained after annealing the starting materials for (a1–a3) $NaFeTiO_4$ and (b1–b3) $Na_{0.75}Fe_{0.75}Ti_{0.25}O_2$ at different times. (**a1, b1**) 2 h, (**a2, b2**) 4 h, and (**a3, b3**) 6 h.

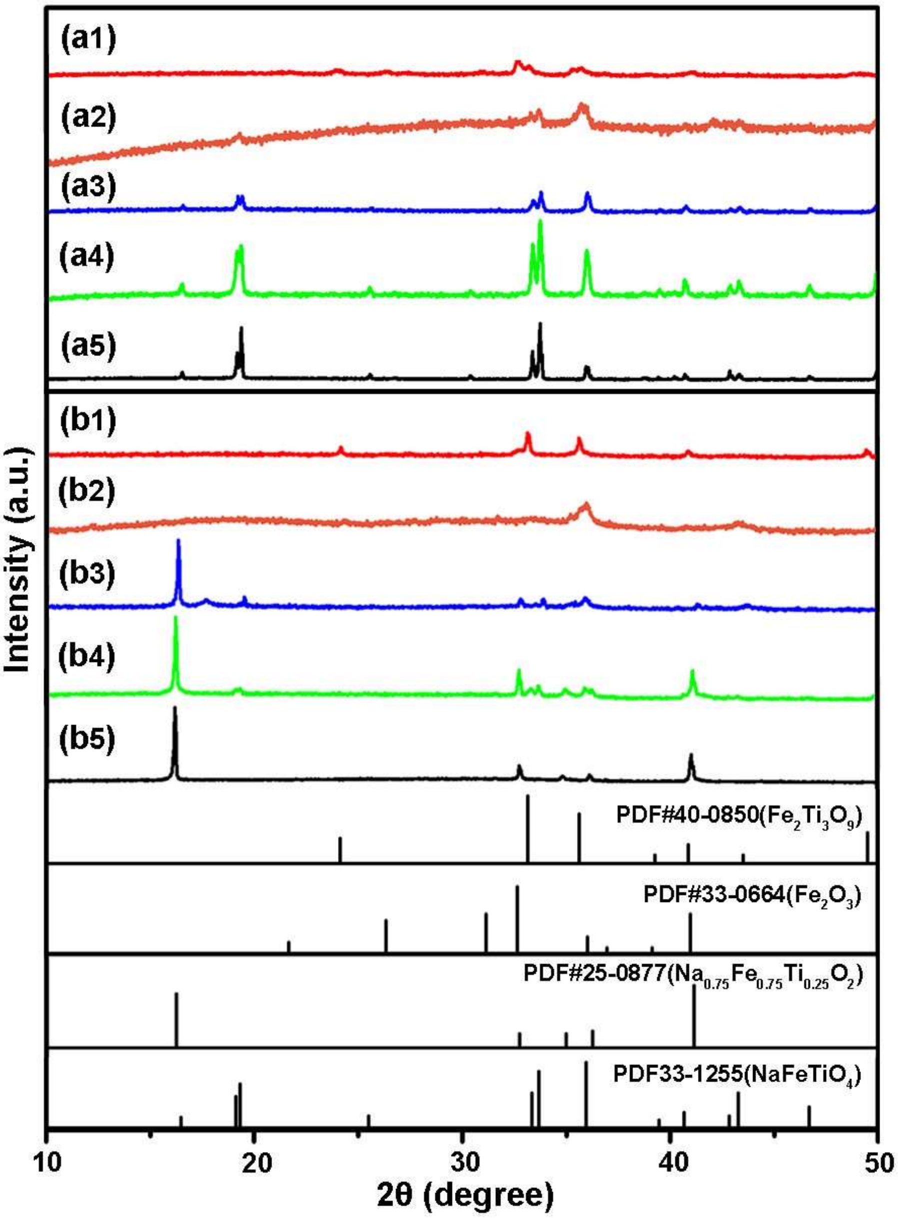

Figure S10. XRD patterns of the products obtained after annealing the starting materials for (a1–a5) $NaFeTiO_4$ and (b1–b5) $Na_{0.75}Fe_{0.75}Ti_{0.25}O_2$ at different temperatures. (**a1, b1**) 600 °C, (**a2, b2**) 700 °C, (**a3, b3**) 800 °C, (**a4, b4**) 900 °C, and (**a5, b5**) 1000 °C.

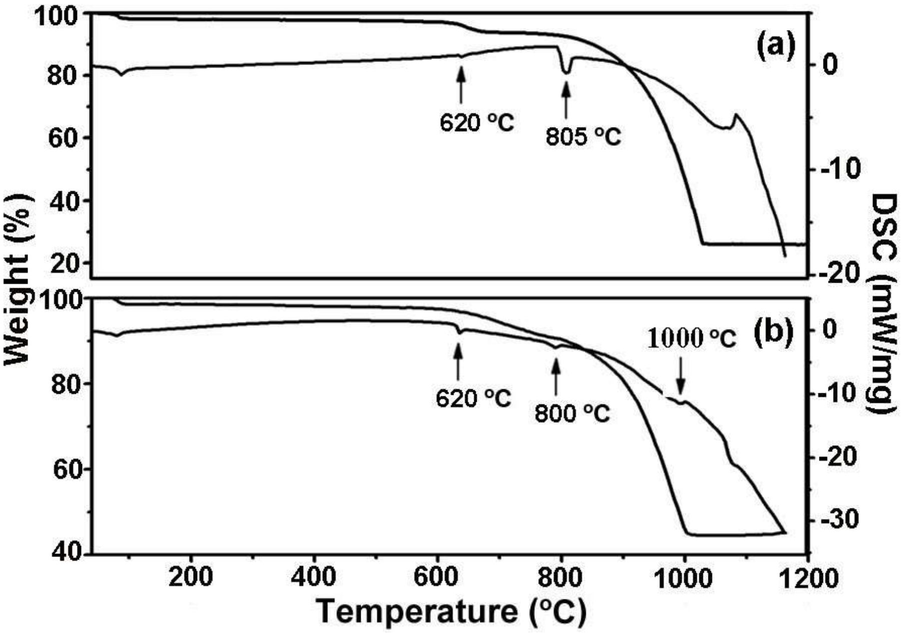

Figure S11. TG-DSC plots of (**a**) $NaFeTiO_4$ needles and (**b**) $Na_{0.75}Fe_{0.75}Ti_{0.25}O_2$ platelets.
